# Enhanced Colon Regeneration in Ulcerative Colitis via Mesenchymal Stem Cell Medium and 17‐β Estradiol

**DOI:** 10.1002/iid3.70387

**Published:** 2026-02-25

**Authors:** Zahra Bakhtiary, Zahra Hosseinpour, Seyyed Meysam Abtahi Froushani

**Affiliations:** ^1^ Department of Histology, Faculty of Veterinary Sciences Ilam University Ilam Iran; ^2^ Department of Histology, Faculty of Veterinary Sciences Islamic Azad University, Babol Branch Babol Iran; ^3^ Department of Microbiology, Faculty of Veterinary Medicine Urmia University Urmia Iran

**Keywords:** 17‐beta‐estradiol, condition medium, histopathology, mesenchymal stem cells, ulcerative colitis

## Abstract

**Background:**

Ulcerative colitis (UC), a form of inflammatory bowel disease (IBD), increases the risk of colorectal cancer. This study investigates the potential of conditioned medium from mesenchymal stem cells (CMm) treated with 17‐beta‐estradiol (E2) to enhance colon regeneration in UC‐affected rats.

**Methods:**

Bone marrow‐derived MSCs from male Wistar rats were cultured with 100 nM E2 for 24 h. UC was induced in three groups: colitis (C), mesenchymal stem cells (MSC), and MSCs + estradiol (MSC + E2). Each group received intraperitoneal injections of 200 µL CMm derived from 2 × 10^6^ cells or E2‐treated CMm three times postinduction. The study evaluated disease activity index (DAI), myeloperoxidase (MPO) levels, nitric oxide (NO), and pro‐inflammatory cytokines (TNF‐α, IL‐1β, IL‐6) in colon tissues, alongside histopathological assessments.

**Results:**

Results showed significant improvements in DAI and reductions in MPO, NO, and cytokine levels in MSC and MSC + E2 groups compared to the C group. Histopathological analysis revealed enhanced colon tissue regeneration in these groups.

**Conclusions:**

These findings suggest that CMm, particularly with E2, may facilitate colonic healing and mitigate inflammation in UC.

## Summary

- A notable decrease in disease symptoms, oxidant levels, and mRNA expression of inflammatory factors was observed in the groups treated with the culture medium of MSCs, especially in those receiving E2.
- Histopathological analysis revealed a significant enhancement in colonic tissue regeneration in both groups administered culture medium after colitis induction.

## Graphical abstract

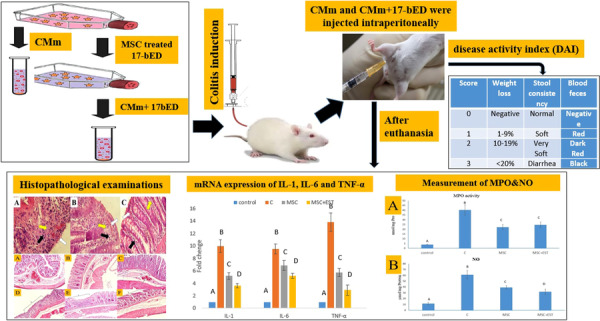

## 1 Introduction

Ulcerative colitis (UC) is a recurrent syndrome and a subgroup of inflammatory bowel disease (IBD) with a high prevalence in the world [1, 2]. The pathogenesis of this disease is unknown, but the disruption of the immune system in tolerance to the gut microbiome and the development of constant inflammation against the natural flora may be one of the reasons for its development [3]. Symptoms of this disease include chronic diarrhea, abdominal pain, the presence of blood and mucus in the stool, loss of appetite and weight loss [4]. Surface erosion of the colon wall begins with bleeding from the rectum (mucosal and submucosal parts) and gradually spreads [5, 6, 7]. In fact, the main inflammation occurs in the rectum and colon, making sufferers susceptible to colorectal cancer [2]. The imbalance between pro‐inflammatory cytokines such as TNF‐α and anti‐inflammatory cytokines such as IL‐1β and IL‐6 in UC plays an important role in modulating inflammation [8]. The use of common E2 and non‐E2 anti‐inflammatory drugs (NSAIDs) for the treatment of IBD [9] has many side effects, including liver, kidney and skin disorders, and so forth [10] and also the lack of adequate response of all patients to the said treatment [11]. A Phase I/II trial used autologous MSCs from adipose tissue in Crohn's disease patients. MSCs were expanded and reinfused, showing safety and potential efficacy in reducing inflammation and improving symptoms, with some patients seeing significant improvements [12]. Moreover, A small trial delivered bone marrow MSCs via endoscopy to treat Crohn's disease, showing clinical improvement and fistula healing in some patients [13]. Of note, the supernatant fluid of mesenchymal cells (CMm) is considered a rich source of paracrine factors such as: VEGF, SCF, HGF, IGF‐1, IGF‐2, and SDF‐1 [14]. By creating a paracrine gradient between the damaged area and the mesenchymal stem cells, these factors cause the mesenchymal cells to be attracted to the damaged organ and thus initiate the process of tissue regeneration. CMm has removed limitations in the use of stem cells for tissue regeneration [15] and plays a role in wound healing [16]. It also leads to an improvement in intestinal inflammatory conditions in rats suffering from colitis by reducing the severity of epithelial cell damage, goblet and crypt cell destruction and inflammatory cell infiltration [17].

17‐beta‐estradiol (E2) reduces intestinal inflammation via the angiotensin II receptor [18]. Thus, several studies have shown the role of E2 in accelerating intestinal mucosal regeneration and reducing intestinal ulceration and bleeding [19] and the role of E2 in reducing inflammatory cell infiltration and damage to the crypts and colonic mucosa of animals in which colitis was induced [20, 21]. E2 receptors are widely distributed on the surface of various cells, such as intestinal mucosa [22] and especially MSCs [23]. E2 typically promotes an M2 anti‐inflammatory macrophage phenotype while inhibiting M1 pro‐inflammatory responses, but the exact effects can vary based on the tissue type, state of inflammation, and the presence of other signals in the microenvironment [24]. E2 receptors (ERs), primarily ERα and ERβ, play significant roles in the physiology of MSCs. E2, through its receptors, can influence the differentiation pathways of MSCs [25]. ERs can activate various signaling pathways, including MAPK/ERK and PI3K/Akt pathways, which play roles in cell survival, proliferation, and differentiation [26]. E2 has immunomodulatory effects, which may influence the ability of MSCs to respond to inflammatory signals. This is particularly relevant in contexts of tissue injury where an inflammatory response is present [27].

The present study therefore aimed to investigate the protective effect of E2‐treated CMm on the process of colon tissue repair in the experimental model of UC.

## 2 Materials and Methods

### 2.1 Isolation and Treatment of MSCs

MSCs were isolated from the tibiae and femora of Wistar rats under anesthesia, with all surrounding soft tissues carefully removed. The bones were flushed with DMEM to collect the cells, which were then centrifuged at 1200 rpm for 5 min, resuspended, and seeded into T‐75 culture flasks at a density of 0.3–0.4 × 10^6^ cells/cm^2^ in low‐glucose DMEM supplemented with 15% heat‐inactivated fetal bovine serum (FBS). Cells were maintained at 37°C in a humidified atmosphere containing 5% CO_2_, with medium changes twice weekly. When cultures reached ~70% confluency, cells were detached using trypsin/EDTA, counted, and subcultured up to the second passage for subsequent experiments.

At Passage 3, cells were untreated (control) or treated with 100 nM E2 for 24 h, after which the medium was removed and replaced with DMEM without FBS for another 24 h. The conditioned medium was collected, centrifuged for 10 min at 300*g*, and filtered with a 0.2‐μm filter to remove debris [27].

### 2.2 Experimental Setup and Animals

For this study, 50 adult male Wistar rats weighing ~200–250 g were obtained from the animal house of Ilam University (Ilam, Iran). The animals were housed under controlled environmental conditions (25°C and 12‐h light–dark cycle) and had free access to standard rodent laboratory diet and water. All animal experiments were conducted in accordance with the Guide for the Care and Use of Laboratory Animals (NIH Publication No. 85‐23, 1996), and the study was approved by the Ilam University Animal Research Ethics Committee (IR.ILAM.REC.1403.023). Ten rats were sacrificed for bone marrow aspiration of MSCs. In total, 40 rats were randomly and equally divided into 4 experimental groups. All groups respectively:

1. Control group: Intrarectal injection of 1 mL normal saline 0.9%,
2. Colitis group (C): Induction of UC by intrarectal injection of 1 mL solution of 4% acetic acid.
3. Mesenchymal group (MSC): After induction of UC, intraperitoneal injection of 200 µL CMm obtained from 2 × 10^6^ MSCs (3 times in 10 days).
4. Mesenchymal + E2 group (MSC + E2): After induction of UC, intraperitoneal injection of 200 µL CMm from 2 × 10^6^ MSCs treated with E2 (3 times in 10 days).

Animals were randomly assigned to four experimental groups using a simple randomization method. All measured parameters were assessed by a single investigator who was blinded to the group allocation. No animals were excluded from the study, and all animals were included in the final analysis.

A completed ARRIVE guidelines checklist is included in Author Checklist (Supporting Information S1). Image of the method summary (Supporting Information S2).

### 2.3 Colitis Induction

Following a 24‐h fasting period, the rats were anesthetized with ether, and a catheter was gently inserted into the rectum. A total of 1 mL of 4% acetic acid solution was administered, after which the animals were held in a diagonal position for 90 s to allow absorption and prevent leakage of the solution [28, 29].

### 2.4 Assessment of the Disease Activity Index (DAI)

All rats were examined daily for changes in body weight, stool consistency, and blood in the stool (DAI) [30]. The values obtained were calculated daily and the overall average DAI was calculated as the Disease Severity Index (DSI) according to Table 1. Also, the Cumulative Disease Index (CDI), which indicates the degree of pain and difficulty suffered by the animal up to the tenth day of the experiment, was calculated by adding the daily DAI to the previous days.

**Table 1:** The Disease Activity Index (DAI) in rats.

Blood feces	Stool consistency	Weight loss	Score
Negative	Normal	Negative	0
Red	Soft	1%–9%	1
Dark red	Very soft	10%–19%	2
Black	Diarrhea	≥ 20%	3

*Note:* DAI was expressed as the sum of scores of all criteria.

### 2.5 MSC Isolation and E2 Treatment

MSCs were isolated from the bilateral thighs and tibias of male Wistar rats according to the established method [27]. The E2 solution was prepared from water‐soluble E2 (Sigma Aldrich, St. Louis, MO, USA) and phosphate‐buffered saline at a final concentration of 100 nM [31], and cells were primed for 24 h. The cells were then washed and maintained for an additional 24 h in medium devoid of FBS and E2. At this stage, the cell supernatant was collected as conditioned medium. Finally, 200 µL CMm derived from 2 × 10^6^ cells treated with E2 were injected intraperitoneally after UC induction.

### 2.6 Measurement of Myeloperoxidase (MPO)

In this method, 10 μL of homogenized colon tissue was mixed with 110 μL of TMB solution (2.9 mM TMB in 14.5% DMSO plus 150 μM sodium sulfate buffer at pH 5.4) and 80 μL of 0.75 μM hydrogen peroxide. After incubation of the samples, 50 μL 2 mM sulfuric acid was added to each chamber and the absorbance was determined using an ELISA Reader at 450 nm [32].

### 2.7 Measurement of Nitric Oxide (NO)

According to the protocol, 50 μL of the Griess reagent (3% phosphoric acid, 0.1% naphthylethylenediamine and 0.1% sulfanilamide) was mixed with 50 μL of a homogenized tissue sample. The resulting mixture was then incubated in the dark at room temperature (25°C) and the optical density was measured at 540 nm using a standard microplate reader [33].

### 2.8 Measurement of mRNA Expression in Inflammatory Cytokines

The level of mRNA expression of tumor necrosis factor‐alpha (TNF‐α), interleukin 1‐beta (IL‐1β), and interleukin‐6 (IL‐6) in the colon samples was monitored by real‐time PCR [30]. Total mRNA was isolated from colon samples using RNX‐Plus solution according to the manufacturer's instructions. The isolated RNA was then used to generate complementary DNA. PCR amplification was performed in triplicate using a SYBR Green kit, following the manufacturer's protocols. The housekeeping gene, GAPDH, was used to normalize data. Forward and reverse primers for mRNA amplification are presented in Table 2. The outcomes were reported as 2^−ΔΔCt^ (mean fold change).

**Table 2:** Real‐time PCR primer sequences.

Primer antisense sequence	Primer sense sequence	
CTTGTTGGCTTATGTTCTG	GGATGATGACGACCTGC	IL‐1 (β)
GGTCCTTAGCCACTCCTTCTGT	CAAAGCCAGAGTCCATTCAGAGC	IL‐6
GAGACTCCTCCCAGGTACATGG	CTTATCTACTCCCAGGTTCTCTTCAA	TNF‐α
GGCATGGACTGTGGTCATGA	CAACTCCCTCAAGATTGTCAGCAA	GAPDH

### 2.9 Histopathological Evaluation

At the end of the treatment period, the animals were euthanized, and the colon tissue was carefully excised and rinsed several times with PBS. The samples were then fixed in 10% buffered formalin for 48 h. Paraffin‐embedded sections of 5–7 μm were prepared and stained with H&E for histopathological evaluation. Inflammatory cells within the intestinal mucosa, eosinophilic cells in the subepithelial layer, and collagen deposition in the mucosal area were quantified within a fixed microscopic field of 0.0625 mm^2^. Vascular congestion in the submucosa and disruption of normal epithelial and crypt architecture were assessed in a fixed field of 1 mm^2^.

A semi‐quantitative scoring system (0–4) was employed to grade disease severity based on the extent and density of inflammatory cell infiltration:

Score 0—Normal: No pathological infiltration. The lamina propria contains only resident cells (e.g., fibroblasts, occasional lymphocytes). Neutrophils and macrophages are absent from extravascular spaces.

Score 1—Minimal (Focal): Mild, localized leukocyte accumulation. Scattered lymphocytes and histiocytes confined to the superficial lamina propria. No submucosal involvement, cryptitis, or architectural disruption.

Score 2—Mild–Moderate (Multifocal): Multifocal leukocyte aggregates in the mucosa, involving over 50% of the lamina propria but sparing the submucosa. Occasional neutrophils present; crypt abscesses and epithelial damage are absent.

Score 3—Moderate–Severe (Diffuse, Submucosal): Dense, diffuse infiltration extending into the submucosa. Cryptitis with or without small crypt abscesses (< 30% of crypts). Neutrophils and macrophages visible; early epithelial erosion may be observed.

Score 4—Severe (Transmural/Pan‐destructive): Dense infiltration throughout the mucosa and submucosa, potentially extending transmurally. Large crypt abscesses (> 30% of crypts), ulceration, or necrosis may be present. Marked architectural disruption with neutrophil predominance.

### 2.10 Statistical Analysis

The data were analyzed using SPSS software, version 23.0 (SPSS Inc., Chicago, IL, USA). For nonparametric values (discontinuous ranks in terms of disease severity), the Kruskal–Wallis test followed by the Mann–Whitney *U* test with Bonferroni was used to compare differences between groups. For continuous data, after testing for normal distribution using the Kolmogorov–Smirnov test, these parametric data were assessed using one‐way analysis of variance (ANOVA) and Dunnett's post hoc test. Results were expressed as mean ± SD. *p* < 0.05 was considered statistically significant.

## 3 Results

### 3.1 Changes in Body Weight

**Figure 1:**
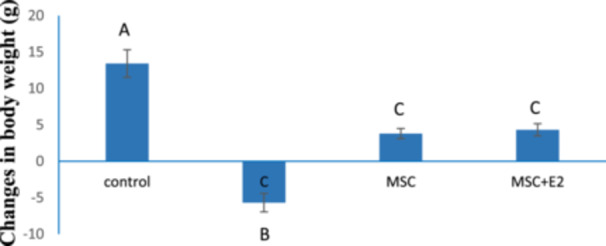
Weight changes of rats in different groups (mean ± SD). The presence of nonsimilar letters indicates a significant difference (*p* < 0.05).

The weight of all rats was calculated as the difference between their weight before the start of the experiment and after the completion of the period. Induction of colitis in the experimental groups resulted in a significant weight loss compared to the control group (13.41 ± 1.89) (*p* < 0.05). However, the MSC (3.79 ± 0.69) and MSC‐E2 (4.32 ± 0.83) groups were able to partially prevent this weight loss and showed a significant difference compared to the C group (−5.68 ± 1.26) (*p* < 0.05) (Figure 1).

### 3.2 Disease Severity Index (DSI)

**Figure 2:**
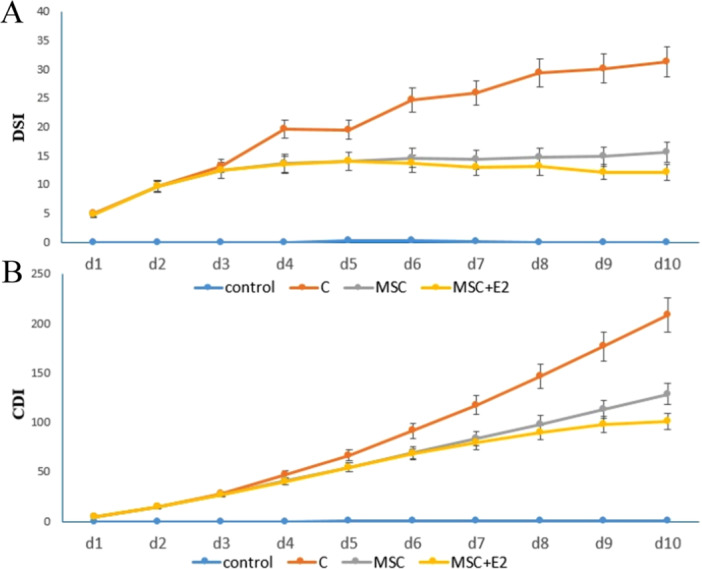
(A) Disease Severity Index in different groups during the first to tenth days, (B) Cumulative Disease Index in different groups during the first to tenth days (mean ± SD).

These results were determined using the symptoms from the DAI index (Table 1) and show the effectiveness of the treatment during the first to tenth day. The DSI on the first to tenth day showed that the changes in this index occurred in the C group from the fourth day and in the MSC and MSC + E2 groups from the sixth day. This means that the treatments (CMm and E2) were able to control the increasing trend of the DSI (Figure 2A).

### 3.3 Cumulative Disease Index (CDI)

The results of this index in Group C show an increasing trend from the fourth day onwards, while in the MSC and MSC + E2 groups it shows a decreasing trend and is lower than in Group C (Figure 2B).

### 3.4 Measurement of Myeloperoxidase (MPO)

**Figure 3:**
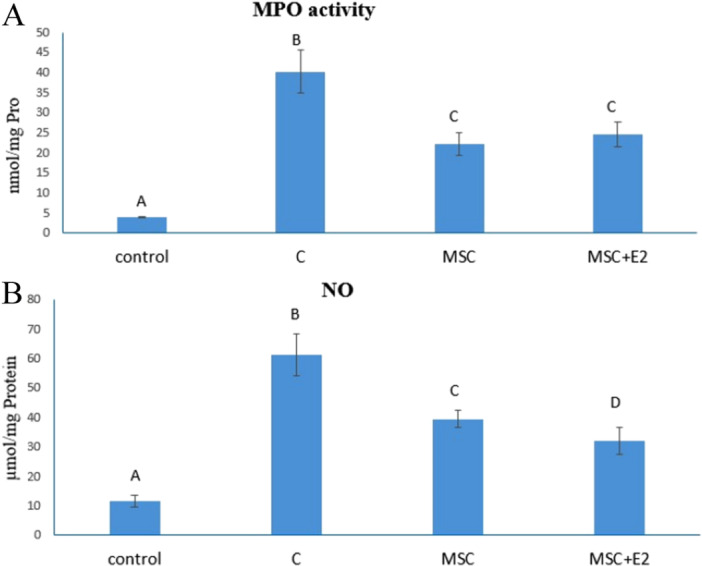
(A) The amount of MPO in different experimental groups. (B) The amount of NO in different experimental groups (mean ± SD). The presence of nonsimilar letters indicates a significant difference (*p* < 0.05).

The results of this study showed a significant increase in MPO in the C group (40.23 ± 5.32) compared to the other groups (*p* < 0.05), while the MSC (22.23 ± 2.77) and MSC + E2 (24.64 ± 3.08) groups were able to significantly reduce this parameter (*p* < 0.05), but there were no significant differences between them (Figure 3A).

### 3.5 Measurement of Nitric Oxide (NO)

The data obtained showed the lowest and highest NO levels compared to the control group (11.5 ± 2.1) in the MSC + E2 (31.89 ± 4.54) and C (61.17 ± 7.19) groups, respectively, which showed significant differences to each other and to other groups (*p* < 0.05). A significant decrease (*p* < 0.05) was also observed in the MSC group (39.34 ± 2.93), but this decrease was not as strong as in the MSC + E2 group (Figure 3B).

### 3.6 Evaluation of the mRNA Expression of IL‐1, IL‐6, and TNF‐α

**Figure 4:**
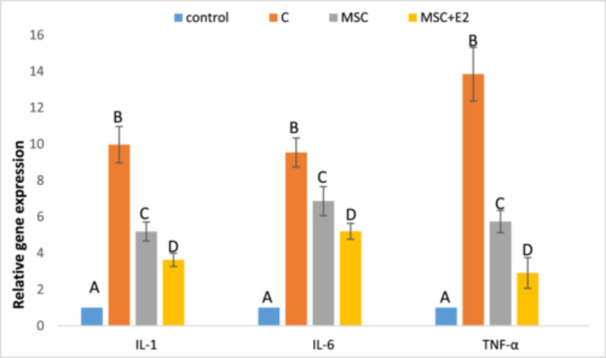
Evaluation of expression levels of IL‐1, IL‐6, and TNF‐α in the colonic homogenate of different groups (mean ± SD) (the housekeeping gene, GAPDH, was used to normalize data). The presence of nonsimilar letters indicates a significant difference (*p* < 0.05).

In this study, the mRNA expression of IL‐6, IL‐1, and TNF‐α was evaluated by real‐time PCR. The results showed that the mRNA expression of all three inflammatory cytokines increased significantly in Group C compared to the other groups (*p* < 0.05). Treatment was able to significantly reduce the mRNA expression of these factors in both the MSC and MSC + E2 groups (*p* < 0.05), but this reduction was more pronounced in the MSC + E2 group, resulting in a significant difference compared to the MSC group (*p* < 0.05) (Figure 4).

### 3.7 Histopathological Examinations

**Figure 5:**
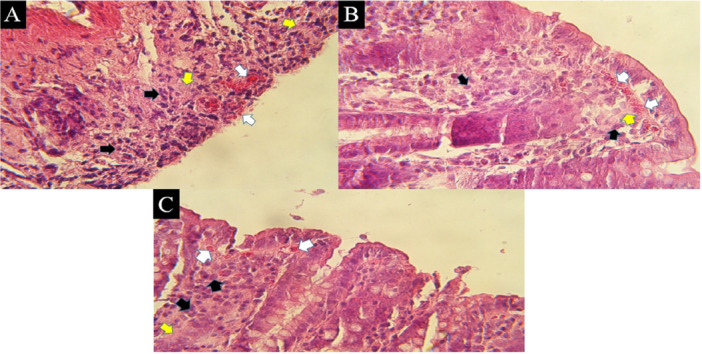
severe leakage of lymphatic cells in the intestinal mucosal layer (black arrows) and eosinophilic cells in the subepithelial surfaces (white arrows) and collagenosis (yellow arrows) in the mucosal area, respectively, in the C (A), MSC (B) and MSC + E2 (C) groups. H&E staining, 400×.

**Figure 6:**
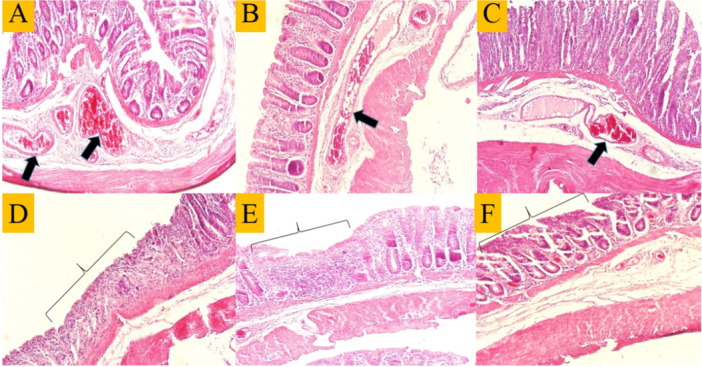
Vascular congestion in the submucosa (arrows) in C (A), MSC (B), and MSC + EST (C) groups and the destruction of the normal structure of intestinal epithelial cells and crypts (square sign) in C (D), MSC (E), and MSC + E2 (F) groups. H&E staining, 100×.

**Figure 7:**
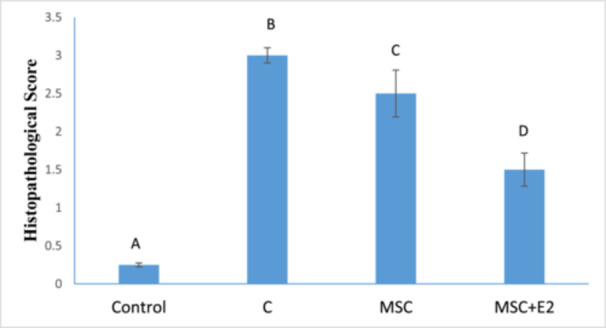
The severity of inflammatory cells was compared on a 5‐point scale from 0 to 4 (mean ± SD). Score 0: normal colon tissue, Score 1: minimal (focal) damage, Score 2: mild–moderate damage (multifocal), Score 3: moderate–severe (diffuse, submucosal), and Score 4: severe damage (transmural/pan‐destructive). The presence of nonsimilar letters indicates a significant difference (*p* < 0.05).

The results of the histopathologic examination of the slides after hematoxylin–eosin staining in the C group showed a strong discharge of lymphatic cells in the mucosal layer of the intestine (the space between the crypts), abundant eosinophilic cells in the subepithelial part of the mucosa and collagenous colitis in the area of the mucosa (Figure 5). Severe vascular congestion in the submucosa and destruction of the normal structure of the intestinal epithelial cells were also observed, particularly in the areas where the intestinal crypts are directly exposed to the lumenal space (Figure 5). Examination of the mucosal area of the MSC group revealed scattered lymphatic cells and, compared to the C group, a decrease in eosinophilic cells as well as collagenous colitis in some areas (Figure 5). Mild to moderate vascular congestion and loss of epithelial cells and crypts were noted in some areas (Figure 6). The evaluation of the slides from the MSC + E2 group also showed a decrease in lymphatic cells, eosinophilia and collagenous colitis compared to the C group (Figure 6). Vascular congestion was less compared to the colitis group and epithelial cell destruction was also observed in some areas (Figure 6). Also, examining the intensity of leukocyte infiltration quantitatively showed a significant decrease in the MSC (2.5 ± 0.3) and MSC + E2 (1.5 ± 0.21) groups compared to the C group (3 ± 0.1) (*p* < 0.05) (Figure 7).

## 4 Discussion

UC is a chronic, progressive condition that profoundly impairs patients' quality of life. For decades, immunomodulatory drugs, including 5‐aminosalicylic acid and glucocorticoids, have been employed to manage UC; however, emerging therapies such as stem cell‐based treatments have recently been proposed [34]. CMm, by maintaining paracrine signaling gradients, enhances the repair of damaged tissue and does not require the stringent sterile conditions necessary for direct mesenchymal cell culture [35]. Additionally, E2 has been shown to reduce apoptosis and promote proliferation of mesenchymal stem cells [31], while also accelerating regeneration and attenuating inflammation in the intestinal mucosa [18, 19]. These findings suggest a potential synergistic effect of E2 and CMm in promoting healing in UC. Recently, it is documented that treatment with a conditioned medium from estradiol‐treated macrophages reduced arthritis severity and improved weight gain in a rat model of rheumatoid arthritis. This was linked to a significant decrease in immune cell proliferation and lower levels of key inflammatory markers and bone‐destructive factors in the blood [36]. Estradiol upregulates the expression of CXCR4 and CCR2 on MSCs. These are receptors that allow MSCs to sense and migrate toward inflammatory signals. This enhances the cells' ability to reach inflamed tissues, a major challenge in MSC therapy [27]. Estradiol treatment increases MSC secretion of VEGF, a critical factor for blood vessel formation and tissue repair [37]. Therapy with estradiol‐primed MSCs leads to a significant decrease in circulating inflammatory markers such as NO and MPO in animal models of MS. In RA rats, this treatment reduced clinical markers like C‐reactive protein and rheumatoid factor to a degree comparable to the drug prednisolone. The results of the changes in body weight of the rats during the test period showed a decrease in the weight of the C group. Treatment with CMm and estradiol in the MSC and MSC + E2 groups accelerated the healing of colon tissue and reduced discomfort. Consequently, these groups experienced significantly less weight loss than the C group. Data from studies on the effect of E2 in ovariectomized female rats after colitis induction also demonstrated reduced weight loss during the experiment [20]. The DSI was used to evaluate treatment efficacy from Days 1 to 10. In the C group, the index began to increase on Day 4, while in the MSC and MSC + E2 groups, changes started on Day 6. Notably, the MSC + E2 group effectively controlled this increase, maintaining the DSI at lower levels than the C group. These findings are consistent with previous studies on the effect of nonadherent mesenchymal cells in induced colitis models in rats [3].

The results of the CDI reflect the level of pain and discomfort experienced by the animals up to Day 10 of the experiment. The upward trend in the C group accelerated from Day 4 compared to the other treatments, whereas the MSC and MSC + E2 groups were able to reduce the index and maintain it at lower levels. In a previous study, tarragon extract (a plant species) reduced CDI in rats with colitis [30]. The combined results of DSI and CDI highlight the role of paracrine factors, including cytokines and growth factors, from CMm in tissue repair [35], as well as the effect of E2 in attenuating intestinal inflammation [18], ultimately improving treatment outcomes in these groups. MPO, a peroxidase enzyme predominantly found in neutrophil granulocytes, generates reactive oxygen species following neutrophil recruitment to acetic acid–induced inflamed colon tissue and plays a key role in inflammation and oxidative stress [30]. Therefore, MPO levels are widely recognized as indicators of acute inflammation [37, 38].

NO is a free radical generated by immune cells following inflammation [30] and contributes to the pathogenesis of UC [39]. Uncontrolled production of reactive species, including NO and ROS, can prolong and exacerbate inflammatory responses [40]. In the present study, MPO and NO levels were highest in the C group and differed significantly from those in the other groups, whereas the MSC and MSC + E2 groups showed a marked reduction in these levels. Notably, the average NO index in the MSC + E2 group was significantly lower than that of the MSC group. Previous studies on UC have reported similar findings, showing decreased MPO levels in animals treated with E2 [41], as well as reductions in MPO and NO levels in groups treated with tarragon extract 30 and both adherent and nonadherent mesenchymal cells [42].

Chronic inflammation promotes tumorigenesis through oxidative stress and the action of pro‐inflammatory factors such as IL‐6 and TNF‐α [43, 44]. An increase in the mRNA expression of inflammatory cytokines in UC rats was an anticipated outcome of the present study. Pro‐inflammatory cytokines, including IL‐6, IL‐1β, and TNF‐α, contribute to the severity of inflammatory responses and tissue damage in IBD [3]. Therefore, reducing levels of these cytokines may represent an effective strategy for managing the clinical symptoms of UC. In this context, Grivennikov and colleagues reported the anti‐inflammatory effects of IL‐1 receptor antagonist (IL1ra) in an acetic acid‐induced UC animal model [45].

In addition, reductions in IL‐1, IL‐6, and TNF‐α levels by adherent and nonadherent bone marrow‐derived mesenchymal cells, tarragon plant extract, and atorvastatin in UC‐induced rats have been reported [3, 30, 46]. This effect may be linked to a decrease in IL‐17, a pro‐inflammatory cytokine that stimulates fibroblasts, endothelial cells, macrophages, and epithelial cells to produce other pro‐inflammatory cytokines, including IL‐1, IL‐6, and TNF‐α [46]. By enhancing paracrine signaling, CMm accelerates the regeneration of damaged tissue [35] and, simultaneously, modulates classical macrophage phenotypes, resulting in decreased production of IL‐6 and TNF‐α and increased secretion of IL‐10. IL‐10 plays a key anti‐inflammatory role by suppressing macrophage and neutrophil activity and inhibiting T cell proliferation, which contributes to chronic inflammation [47]. Two potential mechanisms may explain the efficacy of CMm: it may directly regulate cytokine synthesis by immune cells, or the observed cytokine reduction could be a secondary effect of epithelial repair and tissue regeneration. Nevertheless, further investigation is required to clarify these mechanisms.

The role of E2 in reducing apoptosis and promoting MSC proliferation [31], as well as in accelerating intestinal mucosal regeneration via the angiotensin II receptor, has also been demonstrated [18, 19]. In the present study, histopathological analysis revealed reductions in epithelial cell destruction, infiltration of inflammatory cells, eosinophilia, collagenous colitis in the mucosal layer, and vascular congestion in the submucosa of the colon in the MSC groups, particularly in MSC + E2, compared to the C group. Considering that the generation of oxygen and nitrogen free radicals in damaged tissue compromises epithelial cell integrity [48], the observed decreases in NO and MPO, along with reductions in inflammatory cytokines and DSI in the cell‐treated groups, confirm tissue improvement and enhanced epithelial repair. These findings are consistent with previous studies demonstrating the role of E2 in reducing inflammatory cell infiltration and damage to crypts and colonic mucosa in UC animal models [20, 21].

Recent studies indicate that stem cells and their conditioned media hold significant promise for the treatment of IBD. For example, conditioned medium derived from endometrial regenerative cells has been shown to markedly improve both clinical symptoms and histological alterations in colitis models, highlighting its potential as a cell‐free therapy for UC [49]. Additionally, the utilization of other cell‐free therapies, such as mesenchymal stromal cell‐derived membrane particles (MSC membrane particles), conditioned medium, and mesenchymal stem cell‐derived extracellular vesicles (MSC‐EVs), has been shown to modulate the immune response and attenuate inflammation in IBD, thereby enhancing intestinal tissue repair and regeneration [50, 51]. These findings highlight the therapeutic potential of stem cell‐derived products in managing IBD, although further clinical studies are necessary to establish their efficacy and safety.

## 5 Conclusion

According to the results of the present study, both experimental groups, including MSC and MSC + E2, had positive effects on improving the destructive effects of UC, affecting all measured factors including weight changes, DSI, CDI, MPO, NO, IL‐1, IL‐6 and TNF‐α, as well as all histopathological parameters.

The study indicates that both experimental groups, consisting of MSC and those treated with E2 (MSC + E2), may have beneficial effects in mitigating the harmful impacts associated with colitis. This intervention seems to influence a variety of factors, including weight changes and several inflammatory markers, along with histopathological assessments. It is important to note that while the model used may simulate aspects of UC, the specific context of rat colitis should be acknowledged. Finally, it is important to note that concentrations significantly higher than physiological levels of E2 were utilized in this study, which served solely as a proof‐of‐concept experimental setup. However, this survey is a preliminary study in an animal model, and further studies are required to demonstrate the efficacy of this treatment in humans with UC. It is also necessary to address administration timing, route, and dose–response of estradiol in future research. Of note, the selected dose in this study was chosen according to previous study [27, 31, 36, 37].

### 5.1 Limitations

Although the findings of this study demonstrated encouraging effects of the CMm, particularly when combined with E2, on intestinal improvement in rats with UC, several key limitations warrant careful consideration in the interpretation of the results. The follow‐up period was relatively brief, and only the short‐term therapeutic effects after disease induction were assessed, which precludes evaluation of long‐term treatment outcomes and the sustainability of recovery. This study employed only a single animal model (Wistar rats), potentially restricting the generalizability of the results to specific experimental conditions and posing challenges for direct extrapolation to human subjects. Moreover, the study lacked data on long‐term safety and comprehensive assessment of sustained functional outcomes, such as extended intestinal function or related behavioral measures, which are critical for a complete understanding of the therapeutic potential of CMm. Future research involving longer follow‐up durations, diverse animal models, and focused evaluation of immune and functional outcomes is needed to more thoroughly elucidate the potential effects of this therapeutic approach.

## Author Contributions

**Zahra Bakhtiary:** project administration, supervision, writing – original draft, writing – review and editing. **Zahra Hosseinpour:** project administration, supervision, writing – original draft, writing – review and editing. **Seyyed Meysam Abtahi Froushani:** conceptualization, data curation, investigation, methodology.

## Conflicts of Interest

The authors declare no conflicts of interest.

## Supporting information

S1_arrive.

S2_Image_of_the_method_summary.

## Data Availability

Data will be made available on request.
