# Supplementary figures and images for: Enhanced Colon Regeneration in Ulcerative Colitis via Mesenchymal Stem Cell Medium and 17‐β Estradiol
Source: Immun Inflamm Dis. 2026 Feb 25;14(2):e70387. doi: 10.1002/iid3.70387 (PMC12933408; doi:10.1002/iid3.70387)

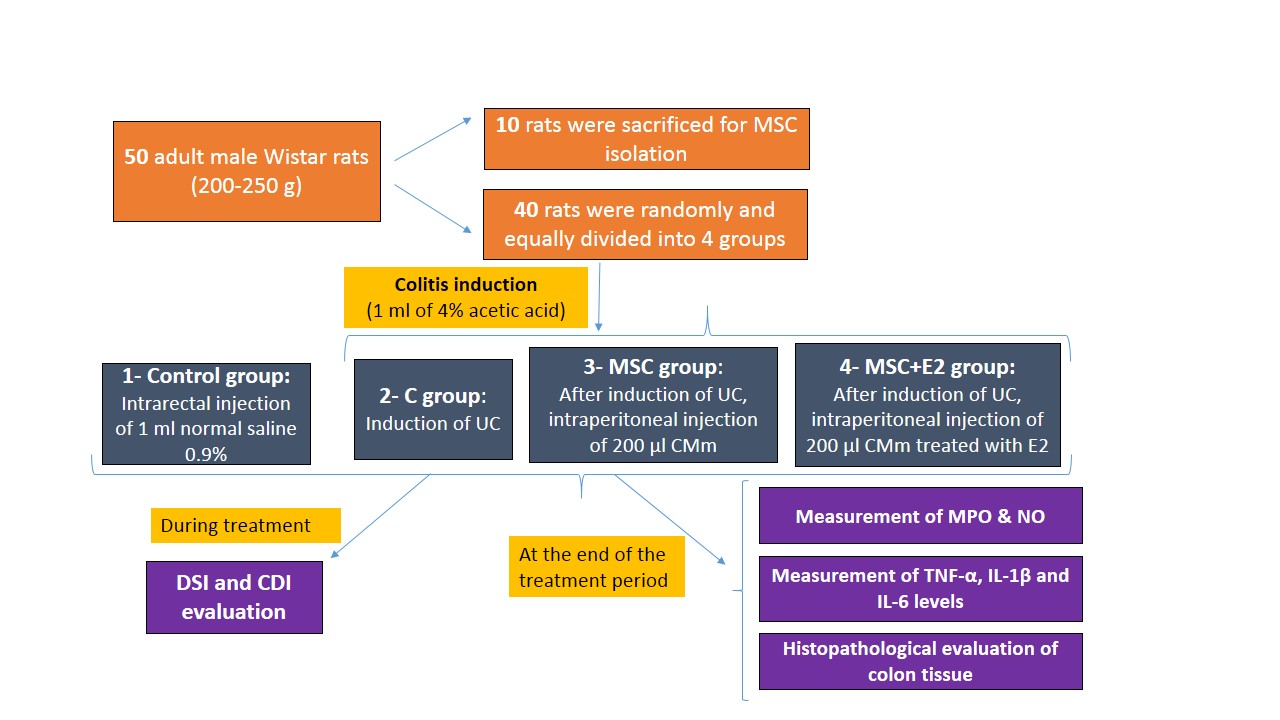


Image of the method summary

Supplement: Supplementary file 2 — S2_Image_of_the_method_summary. [file IID3-14-e70387-s002.docx]
